# Reading the news on Twitter: Source and item memory for social media in younger and older adults

**DOI:** 10.1186/s41235-020-0209-9

**Published:** 2020-03-14

**Authors:** Kimberly A. Bourne, Sarah C. Boland, Grace C. Arnold, Jennifer H. Coane

**Affiliations:** Department of Psychology, Colby College, 4000 Mayflower Hill Drive, Waterville, ME 04901 USA

**Keywords:** Social media, Source memory, Item memory, Aging

## Abstract

**Background:**

Social media content is well-remembered, possibly because of its personal relevance and gossipy nature. It is unclear whether the mnemonic advantage of social media extends to a population less familiar with these platforms and whether knowing the content is from social media sources influences memory. This study examined how the presentation of news-like content in social media affected both item and source memory across two age groups. Younger adults (*n* = 42) and older adults (*n* = 32) studied tweets and news headlines that appeared in the format of Twitter posts or CNN headlines - these items were designed to be either congruent (e.g., tweets formatted as Twitter posts) or incongruent (e.g., tweets formatted as CNN headlines).

**Results:**

For item memory, both age groups correctly recognized tweets more than headlines. Source identification was more accurate when format and content were congruent than incongruent. Signal detection analyses indicated that the source advantage for congruent items was largely driven by a bias to select the format that matched the content’s original source and that this tendency was stronger in older adults.

**Conclusions:**

These results replicate previous literature on the mnemonic advantage of social media content. Although both younger and older adults remembered the content of social media better than the content of news sources, older adults were more sensitive than younger adults to congruency effects in source memory. These findings suggest that older adults rely more on their prior knowledge of conventional language and style in traditional and social media.

## Significance

Social media have become a platform for sharing news, memes, and other content in addition to personal information. Sharing news stories on one’s social media page is easy and common. In addition to remembering *what* one saw, remembering *where* one encountered an event is important. We examined how younger and older adults remember the content and source of items from social media and from traditional news sources. Participants studied news headlines and tweets that were photoshopped to look like CNN-online posts or tweets. Social media content was remembered better than news content, suggesting that the social nature of the material is well-remembered by both younger and older adults. When asked whether studied items had been presented in Twitter format or CNN format, errors revealed that remembering source information is difficult when content and format do not match. Older adults in particular were more likely to assume that a news headline had been studied as a CNN post, even if it had been studied as a Twitter post (and vice versa). In other words, it is easy to misremember where one encountered a piece of information - reading the news on social media might make it harder to remember where the information was actually found. These findings suggest that the erosion of boundaries between news sources and social media has potential implications for remembering the source of information.

## Reading the news on Twitter: source and item memory for social media in younger and older adults

A majority of American adults who use the Internet have social media accounts (e.g., Facebook, Twitter (Greenwood, Perrin, & Duggan, 2016)). Although younger adults are more likely to use social media, use among older adults is increasing (Zickuhr & Madden, 2012). Social media sites are becoming a significant source of news (Hermida, Fletcher, Korell, & Logan, 2012), with 85% of posts on Twitter being headline news or news-like (Kwak, Lee, Park, & Moon, 2010). Thus, how we access the news is evolving, such that traditional news sources, like newspapers, cable, and radio, are becoming less discernible from social media. With the blending of traditional and social media, it is important to assess the extent to which one can accurately recall both the content and the source of information encountered in these media and whether the age of the user matters.

In the present study, younger and older participants studied social media posts (i.e., tweets) and news headlines selected from CNN online that were formatted to appear as items on a Twitter feed or news items on CNN. Item and source memory tests were administered to examine how content and perceived source were remembered. Given the blending of news and social media platforms and the ease with which information can be shared across platforms (e.g., linking a news story on Facebook or Twitter) it is worthwhile to investigate memory differences for social media and news across ages. In this study, we were particularly interested in situations when the content does not match the format of the platform (e.g., a news item processed on social media) and how source memory may be affected if readers are accessing news across multiple sites.

Source memory involves the recollection of details and context surrounding the encoding of an event (e.g., Johnson, Hashtroudi, & Lindsay, 1993). The ability to accurately recall where one learned something is fundamental for evaluating its veracity, or for finding the information at a later date. Relative to item memory, which can be largely familiarity-driven, source memory involves more controlled and recollective processes because it requires binding episodic details. The integration of news on social media (Kwak et al., 2010) might result in source confusion. For example, people misidentified televised news-like advertisements as actual news about 70% of the time (Yegiyan & Grabe, 2007). Thus, people reading news on social media might fail to remember that news-like information presented on social media websites was not actually news.

Factors such as a person’s age and their familiarity with the content can affect the accuracy of source memory. Because of older adults’ deficits in source and associative memory (Old & Naveh-Benjamin, 2008) and their increasing rate of interactions with social media, the proliferation of news items on social media may negatively affect their memory. On episodic memory tests, older adults frequently show deficits in memory relative to younger adults, most noticeably when the tests require use of controlled processes (Balota, Dolan, & Duchek, 2000; Craik & Byrd, 1982; Ferguson, Hashtroudi, & Johnson, 1992). Younger and older adults’ item memory can be similar, especially when the task offers environmental support (e.g., recognition tests), but older adults perform worse in source memory tests (Balota et al., 2000; McIntyre & Craik, 1987; Spencer & Raz, 1995). These deficits in source memory may be attributed to a lack of detail-specific encoding. Johnson et al. (1993) hypothesized older adults were less likely to encode specific contextual details about the to-be-remembered items. Rather, they encode in a more general, automatic manner resulting in poorer retrieval cues and overall impairment in source memory (Rabinowitz, Craik, & Ackerman, 1982). In some cases, however, older adults had better source memory for conceptual details (i.e., whether the speaker was lying or telling the truth) than perceptual details (i.e., the gender of the speaker; Rahhal, May, & Hasher, 2002). According to socio-emotional selectivity theory (Carstensen, 1995), how individuals handle socially relevant interactions is adaptive and dependent on life stages. Older adults in particular might shift their attention away from knowledge-gathering and focus more on processes such as emotion regulation that enhance wellbeing. This shift in focus can then facilitate performance on tasks that emphasize emotional or pro-social processing. For example, older adults tend to remember emotionally and personally relevant information relatively well, and may even compensate for deficits in source memory when given socio-emotional information that is consistent with their current goals. In one study (Rahhal et al., 2002), older adults remembered the source of information as well as younger adults when they were given information about the speakers (e.g., speaker A lies, speaker B tells the truth), suggesting that the socio-emotional information provided allowed them to encode the information better.

Even when the material is not personally relevant or emotional in content, familiarity in some domains may help older adults compensate for deficits in processing resources (Park, 1997). Specifically, older adults have an elaborate semantic memory, that often exceeds that of younger adults (e.g., Salthouse, 2004) and can enhance episodic retrieval. If older adults are able to rely on prior knowledge or capitalize on their experiences, age differences can be reduced or eliminated (see Umanath & Marsh, 2014 for a review). In one study (Matzen & Benjamin, 2013), older adults outperformed younger adults in memory for sentences, a finding the authors attributed to the added experience in reading acquired over a lifetime.

In addition to increased language knowledge and experience, older adults also tend to rely more than younger adults on schemas and schematic knowledge (i.e., stored knowledge that organizes information and specifies the relations between elements). Stereotypes are one form of schematic knowledge. For example, older adults have been shown to be more sensitive to stereotype-consistent information than younger adults, such that the two age groups were equally accurate at attributing stereotype consistent information to the proper source, but older adults were less accurate on stereotype inconsistent content (Mather, Johnson, & De Leonardis, 1999). Even when learning new information, older adults show sensitivity to the compatibility between elements of the stimuli. Specifically, compatible pairings of brand logo graphics and brand names (e.g., pairs that were inherently related) improved associative memory in both younger and older adults and, importantly, eliminated age-related deficits relative to incompatible pairings (Mohanty, Naveh-Benjamin, & Ratneshwar, 2016).

Thus, although older adults frequently do exhibit deficits in source memory, there are circumstances that mitigate these deficits. Specifically, relatedness, prior knowledge, and schematic knowledge can moderate age-related differences in performance. Not surprisingly, younger adults also frequently benefit from these factors as well, indicating that both age groups should benefit when to-be-remembered material is compatible with its source. In the present context, older adults presumably have more experience than their younger counterparts with traditional news media, such as newspaper, radios, and television. Although older adults are increasingly accessing online sources, they still tend to prefer to follow news in print format (Mitchell, 2016). Importantly, their familiarity with sources such as CNN is likely to be quite high. However, social media platforms, such as Twitter, may not be as familiar to older adults. Therefore, they may not be able to capitalize on prior knowledge and integrate this information into existing systems.

The congruency of content and source may also affect older adults’ recollection. Because of older adults’ increased knowledge base and tendency to rely on schematic or semantic knowledge, source memory might be disrupted if the format (which indicates source) does not align with the content (e.g., if a news headline appeared as a Twitter post). If older adults have a richer knowledge of what items are “news” items, seeing these items in the context of a tweet might be particularly disruptive. In other words, when the source does not align with the content, it may become more difficult for older adults to identify the correct source, as they can no longer rely on the integrative nature of a congruent manipulation (e.g., a tweet that appears as a Twitter post).

Credibility is another factor that may impact source memory. Older adults who rate social media posts as less credible or reliable may not devote the processing resources required during the encoding phase to compensate for the age-related deficits. Mutter, Lindsey, and Pliske (1995) examined the effect of repetition on truth judgments (in which repeated statements are rated as more true than non-repeated statements, regardless of statement veracity) in younger and older adults. Older adults were less accurate at recognizing the source of not credible items than the source of credible items. More generally, given the link between attention and memory (Mulligan, 2008), people might have poor memory for social media because it may be perceived as unimportant and they thus devote fewer attentional resources to this content.

Nevertheless, Mickes et al. (2013) found that younger adults had a robust memory for social media content. In their study, college-aged participants were presented with faces, book sentences, or Facebook posts. Participants remembered Facebook posts better than faces and excerpts from books, a finding Mickes et al. attributed to the personal, socially relevant nature of the content. In another experiment, headlines, sentences, and comments were selected from the Entertainment or Breaking News sections of CNN Twitter feeds. People recognized ttems from CNN’s entertainment section better than those from CNN’s news section, suggesting that the social quality of the information - be it the gossipy nature of entertainment news or status updates on Facebook - contributed to memorability. Thus, Mickes et al. concluded that content from social media was remembered better than other types of content, a fact they attributed to the gossipy nature of the information and to its reliance on natural language.

However, Mickes et al. (2013) did not examine the impact of knowing the source of the items. In their study, all items were presented without any source identifiers (i.e., as typed sentences). Including the source of the information - by presenting a social media post in its original format, with a user profile picture, and date and time information - might affect the item’s memorability. Raj and Bell (2010) suggest that binding the contextual information and content enhances the recollection of complex memories. Inclusion of additional formatting information might also increase the level of detail or distinctiveness (Hunt & McDaniel, 1993), thereby improving memory. Although older adults retained less source information than younger adults in a study examining memory for news sources (i.e., print, radio, and TV (Frieske & Park, 1999)), both groups performed better on source tests for richer content, suggesting that the additional detail provided by the formatting might increase performance. Alternatively, familiarity and source credibility may negatively affect the extent to which material is remembered. For example, Lucassen and Schraagen (2013) found that when college-aged students were presented familiar versus unfamiliar material in either a Wikipedia or non-Wikipedia format (i.e., source information was not provided), familiar material was considered less credible when presented in the Wikipedia format, although there was no effect for unfamiliar material. Therefore, if an item is recognized as coming from a social media or user-generated source, it might be considered of less importance or less reliable and thus not be processed as deeply.

In the present study, we extended Mickes et al.’s (2013) findings by including an older adult sample and by examining source memory. The aging sample allowed us to address questions such as the importance of prior experience with social media, the role of controlled processes (Craik & Byrd, 1982), and the role of schematic/semantic support (Umanath & Marsh, 2014). Participants studied items from Twitter and CNN that were digitally altered to appear as tweets or as headlines. We assessed item and source memory for congruent (e.g., tweets formatted as Twitter posts) and incongruent (e.g., tweets formatted as CNN headlines) items. This design allowed us to examine whether the appearance or format of to-be-remembered information, which indicates its source (i.e., Twitter or CNN), contributes to memory above and beyond content and whether congruency between content and format further affects performance.

If the content, specifically the socially relevant nature of social media, is driving memory, tweets should be remembered better than headlines, regardless of format, consistent with Mickes et al. (2013). According to predictions from socio-emotional selectivity theory (Carstensen, 1995), older adults might also show a mnemonic advantage for tweets because of the social content provided by these items. Alternatively, if the social media advantage is driven by content familiarity, older adults might fail to show a mnemonic advantage, due to reduced schematic or semantic support for the content (Umanath & Marsh, 2014). Thus, whereas we expected to replicate the social media advantage in younger adults, predictions about older adults’ performance were less clear. However, if social media sources are deemed as less credible (Lucassen & Schraagen, 2013) and thus devoted fewer attentional resources during encoding (Mutter et al., 1995), then inclusion of the formatting information might eliminate the mnemonic advantage for social media content in both age groups.

Regarding source identification processes, due to older adults’ deficits in source memory coupled with lower familiarity with the platform and possibly the content of the tweets, we expected younger adults to identify the source accurately more often than older adults (Balota et al., 2000), primarily when the stimuli were incongruent. Congruent stimuli were expected to enhance source memory for both younger and older adults, as suggested by prior work showing improved source memory for compatible over incompatible information in both age groups (Mohanty et al., 2016). Given the increased reliance in older adults on factors such as compatibility and relatedness, compared to younger adults, we expected the older participants to be especially challenged when remembering the source of incongruent items.

## Method

### Participants

Undergraduate students (*n* = 42; 64% women) from Colby College and healthy older adults (*n* = 32; 72% women) from the surrounding area participated in the study.^1^ Older adults were healthy, independent, community-dwelling participants who arranged their own transportation (see Table 1). Older adults had more years of education than younger adults, *t* (34.17) = 4.92, *p* < .001 (degrees of freedom reflect a correction because of unequal variance between samples). Younger adults were compensated with candy and an opportunity to earn a $5 gift card through a raffle draw. Older adults were compensated at a rate of $10 per hour. The study was approved by the Institutional Review Board at Colby College. The minimum sample size was determined based on the observed effect size calculated from experiment 1 in Mickes et al. (2013). Using the means reported in their paper, we obtained an estimate of *d* of 1.22 based on a between-participants design; calculations in G*Power 3.1 (Faul, Erdfelder, Buchner, & Lang, 2009) indicated a sample size of 11 was necessary to obtain 0.96 power in a within-subjects design.^2^

**Table 1:** Participant age and education as a function of age group

	Mean	SD	Range
Younger adults			
Age, years	20.02	1.09	18–23
Education, years	13.38	.80	12–15
Older adults			
Age, years	70.89	6.30	63–94
Education, years	16.13	3.08	12–22

Based on responses to a questionnaire, described In the Materials, all younger adults reported using social media (e.g., Twitter, Facebook) compared to 53% of older adults. Of those who reported using social media, 74% of younger adults used social media 1–5 h a day, whereas 53% of older adults used social media 0–2 h per day. About 19% of younger adults reported reading the news daily compared to 78% of older adults. Frequency of social media and news use (e.g., daily, weekly) were converted to numerical values, with higher values reflecting more use. Frequency of reading the news was on a 5-point scale (1 = less than once a month, 5 = daily) and frequency of social media use was on a 5-point scale (1 = less than once a day, 5 = more than 13 times a day).^3^ Older adults read the news (*M* = 4.47, *SD* = 1.16) more than younger adults (*M* = 3.33, *SD* = 1.26), *t* (72) = 3.96, *p* < .001, 95% CI [0.56, 1.71], *d* = 0.94, whereas younger adults used social media (*M* = 3.36, *SD* = .93) more frequently than older adults (*M* = 1.83, *SD* = 0.78; *t* (58) = 6.06, *p* < .001, 95% CI [− 2.03, − 1.02], *d* = 1.78). Thus, older adults were less likely to use social media than younger adults and those who did use it tended to do so less often. Conversely, younger adults read the news less often than older adults.

### Materials

**Fig. 1:**
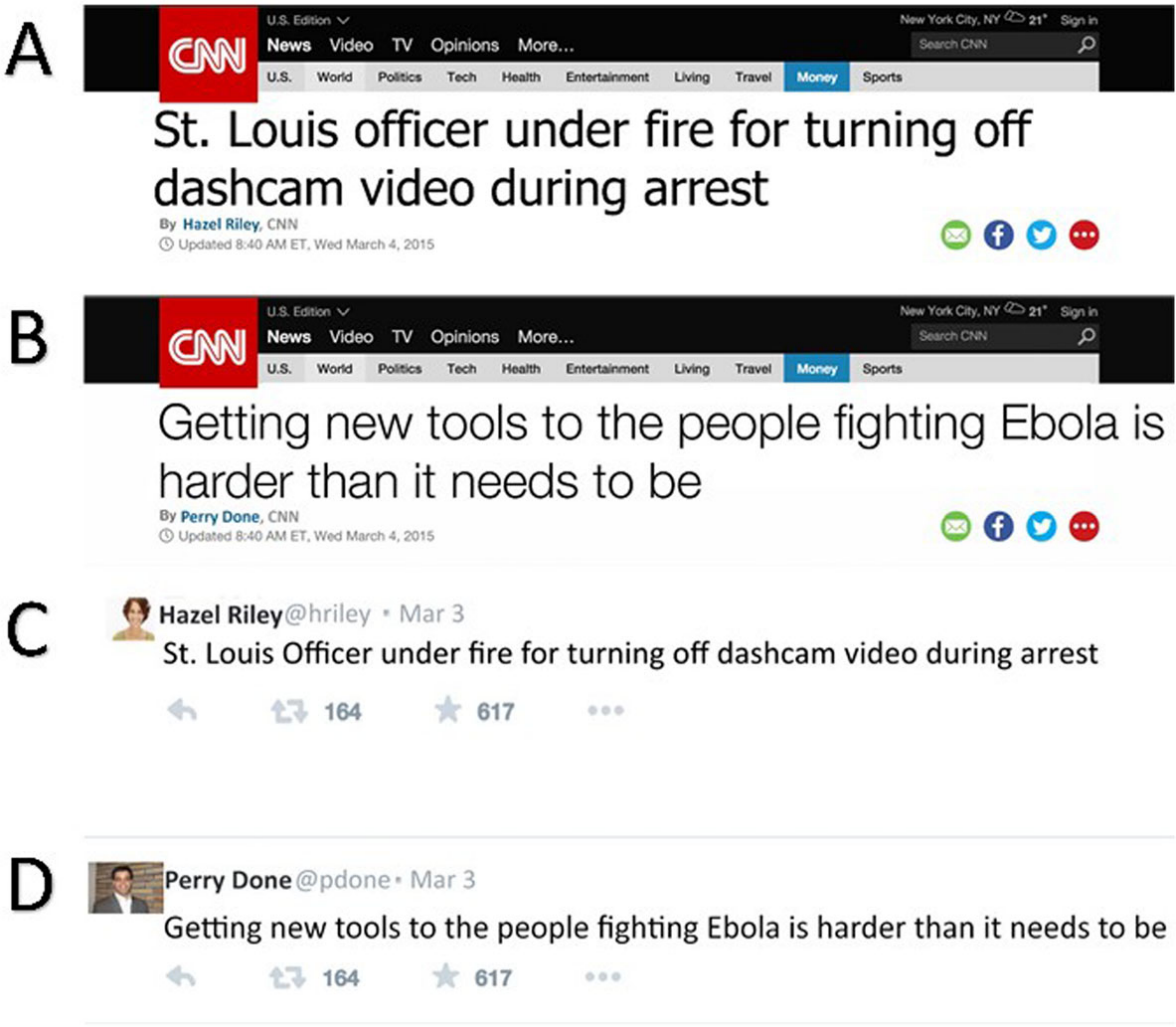
Sample stimuli. **a** Headline formatted as a CNN news item. **b** Tweet formatted as a CNN news item. **c** Headline formatted as a Twitter post. **d** Tweet formatted as a Twitter post

We selected 80 headlines from an online news source, CNN, and 80 tweets from the social media website, Twitter (see Table 2 for sample stimuli). There was a large range of topics for both tweets and headlines (e.g., entertainment, politics, health, sports, travel, food). Tweets were selected from public accounts. Tweets and headlines were between 30 and 100 characters in length, with headlines averaging 56.23 characters (*SE* = 1.44) and tweets averaging 61.03 characters in length (*SE* = 2.13), with minimal to no hashtags or links. Tweets were slightly longer than headlines (*p* = .064). All identifying content (i.e., names, handles, and pictures) were removed, and replaced with names from an online random name generator (http://random-name-generator.info/) and headshots selected online from public sources. The same names as CNN bylines and as Twitter user names were used. The format (CNN and Twitter background) and content (headline and tweet) were factorially crossed to create four conditions: congruent (i.e., tweets presented on a Twitter background and CNN headlines presented on a CNN background) or incongruent (i.e., tweets on a CNN background and CNN headlines on a Twitter background; see Fig. 1). Content in the CNN format was typed in size 115 Tahoma font. Content in the Twitter format was size 36 Calibri font to mimic the actual appearance of these items in a naturalistic setting. Content was formatted using photo-editing software (Adobe Photoshop).

**Table 2:** Sample headlines and tweets

Headline	Tweet
Decline in smoking rates could increase deaths in lung cancer	Amazing. Beck and Chris Martin sure did … stand there super well. So much Energy!
Sean Penn and others rile up social media at the Oscars	Shia Lebouf sure knows how to read a teleprompter
Hush! There’s a secret bar inside this bar Space	North Carolina still has my heart after all these years
Divided House GOP turns to special rule to pass budget	It’s official. Katy Perry is magic. Space filler space filler
Work/life balance an impossible dream? Space filler	So sad to hear the news from Pakistan. Rest in peace

For the final recognition test, all headlines and tweets were typed in size 12 black Times New Roman font on white background and all contextual information, such as background, formatting, names and faces, was removed. Stimuli were counterbalanced across conditions (congruent and incongruent) and study status (studied versus non-studied), yielding four different scripts. An approximately equal number of participants were tested in each experimental script. The full set of stimuli is available in the Additional file 1.

We also created a brief questionnaire on news and social media use. Participants indicated what type of social media they used, how often they checked social media, the source of their news, and how often they checked the news.

### Procedure

Participants were tested individually. The experiment was programmed using the Qualtrics platform (Qualtrics, 2015). Participants were instructed to study the stimuli for an unspecified memory test. Next, participants completed a brief demographic questionnaire and the study phase: 80 items, 20 from each of the four conditions, were presented one at a time in random order. Because older adults have slower processing times (Bäckman, 1986; Salthouse, 2004), younger adults saw each item for 3 s, whereas older adults saw each stimulus for 5 s to account for potential performance deficits due to age-related slowing (Hay & Jacoby, 1999; Lemke & Zimprich, 2005). Participants then completed the social media and news questionnaire. Afterwards, they completed the item recognition test, which consisted of the 160 stimuli, 80 studied and 80 new, presented one at a time in random order. If participants identified an item as “old”, they indicated whether the statement had been presented as a Twitter post or as a CNN headline. Testing was self-paced and took approximately 15 min for younger adults and about 30 min for older adults. When participants finished, they were thanked for their time, debriefed, and compensated.

## Results

Alpha was set at .05. When required, degrees of freedom reported are corrected for violations of assumptions and Bonferroni corrections are applied to pairwise comparisons.

### Item memory

To analyze item recognition, we conducted analyses in line with Mickes et al. (2013). We calculated *d’* scores as a measure of discriminability (see Table 3), using the formulae in Macmillan and Creelman (2005). Hit rates of 1.0 and false alarm rates of 0 were transformed using 1 – ½ N and ½ N, respectively. For each type of item generated by factorially crossing content and format, we calculated *d’* by using the hit rate obtained and the false alarm rate for non-studied foils with the same content (e.g., for tweets presented as CNN headlines, we used the false alarm rate to non-studied tweets). Analyses on raw accuracy are reported in Additional file 1.

**Table 3:** Proportion of “old” responses and signal detection estimates as a function of age, item content, and item format

	Formatting at encoding		Non-studied	*d’*	
	Congruent	Incongruent		Congruent	Incongruent
Younger adults					
Headlines	0.76 (0.03)	0.76 (0.03)	0.09 (.02)	2.40 (0.15)	2.44 (0.15)
Tweets	0.82 (0.03)	0.82 (0.02)	0.09 (.02)	2.60 (0.15)	2.62 (0.15)
Older adults					
Headlines	0.76 (0.03)	0.75 (0.03)	0.13 (.03)	2.23 (0.17)	2.18 (0.17)
Tweets	0.79 (0.03)	0.83 (0.03)	0.13 (.02)	2.29 (0.17)	2.49 (0.17)

Standard error presented in parentheses

We conducted 2 (age) × 2 (content: headline and tweet) × 2 (format: CNN and Twitter) mixed analysis of variance (ANOVA) on the *d’* scores. Age was a between-subjects variable and content and format were within-subject factors. Tweets (*M* = 2.50, *SE* = 0.11, 95% CI [2.28, 2.71]), regardless of format, were recognized better than headlines (*M* = 2.31, *SE* = 0.11, 95% CI [2.10, 2.53]), *F*(1, 72) = 6.52, *p* = .013, partial *η*^2^ = 0.08. No other effects were reliable, all *F* values < 1.81, all *p* values > .18. In summary, we replicated Mickes et al.’s (2013) finding of enhanced memory for social media posts relative to traditional news items in younger and older adults.

### Source identification

We defined source identification as correctly selecting the format in which an item was studied. Thus, correct source identification required retrieving specific details about the encoding event, such as recollecting that a statement was associated with the Twitter logo or with CNN’s logo. To analyze source identification, 2 (age) × 2 (content) × 2 (format) mixed ANOVA was conducted on the proportion of correct source responses (e.g., correctly saying “CNN” as the source of a tweet studied as a CNN post). Older adults (*M* = 0.58, *SE* = 0.02, 95% CI [0.54, 0.61]) performed worse overall than younger adults (*M* = 0.72, *SE* = 0.02, 95% CI [0.69, 0.75]), *F*(1, 72) = 32.02, *p* < .001, partial *η*^2^ = 0.31. The source of tweets (*M* = 0.67, *SE* = 0.01, 95% CI [0.64, 0.70]) was correctly identified more than the source of headlines (*M* = 0.62, *SE* = 0.02, 95% CI [0.60, 65]), *F*(1, 72) = 12.43, *p* = .001, partial *η*^2^ = .015. Three higher order effects were reliable: the content by age interaction, *F*(1, 72) = 10.09, *p* = .002, partial *η*^2^ = 0.12, the content by format interaction, *F*(1, 72) = 124.46, *p* < .001, partial *η*^2^ = 0.63, and the three-way interaction between age, content, and format, *F*(1, 72) = 39.02, *p* < .001, partial *η*^2^ = 0.35. No other effects were significant, all F values < 0.76, all *p* values > .39.

For the sake of brevity, we focus on the three-way interaction (analyses on the two lower-order interactions are reported in Additional file 1). We conducted two 2-way ANOVAs to separately examine the effects of content and of format across ages. Younger adults had better source identification for tweets than headlines, *F*(1, 41) = 20.96, *p* < .001, partial *η*^2^ = 0.34. The effect of format was not significant, *F*(1,41) = 0.32, *p* = .57, partial *η*^2^ = 0.01, but there was a significant interaction between content and format, *F*(1, 41) = 15.95, *p* < .001, partial *η*^2^ = 0.28. The source of headlines was better recognized when presented in the CNN format than in the Twitter format, *t*(1, 41) = 3.69, *p* = .001, 95% CI [0.07, 0.24], *d* = 0.55, and the source of tweets was better recognized when presented in the Twitter format than in the CNN format, *t*(1, 41) = − 2.78, *p* = .008, 95% CI [− 0.22, − 0.03], *d* = 0.44. In summary, younger adults showed a source identification advantage for social media content and a large congruency effect.

Older adults showed no effect of content or format, *F* values < .39, *p* values > .54 for both. The interaction was significant, *F*(1, 31) = 114.30, *p* < .001, partial *η*^2^ = 0.79. Older adults better identified the source when the format and content were congruent; this occurred for both headlines, *t*(1,31) = 6.88, *p* < .001, 95% CI [0.33, 0.60], *d* = 1.23, and for tweets, *t*(31) = − 7.88, *p* < .001, 95% CI [− 0.66, − 0.39], *d* = 1.38. As is evident in Table 4, older adults’ source accuracy was no different to that of younger adults for congruent items (the difference was not significant for headlines or tweets, both *p* values > .24). However, for incongruent items, older adults showed a significantly marked deficit in accuracy (both *p* values < .001). Thus, the three-way interaction indicates that older adults were more affected by the incongruency than younger adults were - they had similar performance on congruent items but markedly decreased performance on incongruent items.

**Table 4:** Proportion of sources correctly identified and signal detection parameters as a function of age, item content, and item format

	Formatting at encoding		Non-studied	*d’*	
	Congruent	Incongruent		Congruent	Incongruent
Younger Adults					
Headlines	0.75 (0.03)	0.60 (0.03)	0.55 (0.06)	1.08 (0.13)	1.09 (0.13)
Tweets	0.83 (0.03)	0.70 (0.04)	0.75 (0.05)	1.71 (0.13)	1.71 (0.13)
Older Adults					
Headlines	0.81 (0.04)	0.34 (0.04)	0.80 (0.06)	0.54 (0.15)	0.55 (0.15)
Tweets	0.84 (0.03)	0.32 (0.04)	0.89 (0.06)	0.60 (0.15)	0.61 (0.15)

Standard error is presented in parentheses

To further explore the factors driving the large age effect as a function of congruency we calculated *d’* scores 4. For hit rates we used correct source responses to congruent studied items (e.g., the proportion of correct “CNN” source responses for headlines formatted as CNN headlines) and for false alarms, we used incorrect source responses for incongruent studied items (e.g., the proportion of incorrect “CNN” source responses for headlines formatted as tweets). In other words, we calculated *d’* for source identification by holding source response constant to determine whether the effect was driven by a bias^5^ to respond with one source or the other. Scores of 1.0 and 0 were corrected as described above.

**Fig. 2:**
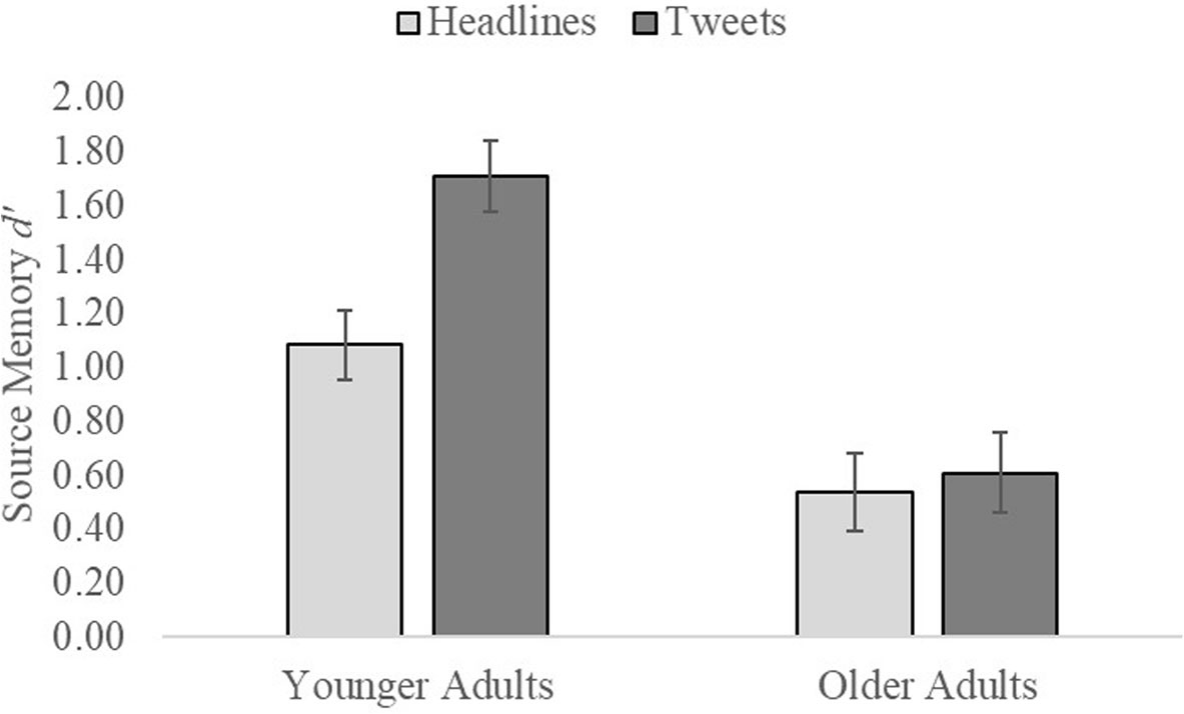
Source memory *d’* as a function of item content (headlines versus tweets) and age

We conducted a 2 (age) × 2 (content) × 2 (format) mixed ANOVA (see Fig. 2) on the *d’* scores. Overall, younger adults (*M* = 1.40, *SE* = 1.11, 95% CI [1.17, 1.62]) had better source memory than older adults (*M* = .57, *SE* = .13, 95% CI [.31, .83]), *F*(1, 72) = 23.08, *p* < .001, partial *η*^2^ = 0.24. The source of tweets (*M* = 1.16, *SE* = 0.10, 95% CI [0.96, 1.35]) was accurately identified more than the source of headlines (*M* = 0.82, *SE* = 0.10, 95% CI [0.62, 1.01]), *F*(1, 72) = 12.67, *p* = .001, partial *η*^2^ = 0.15. These main effects were qualified by a content-by-age interaction, *F*(1, 72) = 8.52, *p* = .005, partial *η*^2^ = 0.11. Older adults showed no difference between source memory for tweets and headlines, *t*(31) = − 0.43, *p* = .67, 95% CI [− 0.35, 0.23], *d* = 0.08. Conversely, younger adults had better memory for the source of tweets (*M* = 1.71, *SE* = 0.13), than for the source of headlines (*M* = 1.09, *SE* = 0.13), *t*(41) = − 4.89, *p* < .001, 95% CI [− 0.88, − 0.36], *d* = 0.75. These analyses suggest that in older adults the congruency effect was partially driven not by better source memory, but by a strong bias to respond with a given format on the source test based on the content of the item rather than item-specific memory for the study event (e.g., if it sounds like a tweet, assume it originally appeared on a Twitter background). When we accounted for response bias (i.e., incorrectly responding “CNN” for the source of a headline formatted as a tweet), older adults showed worse source memory than younger adults. In sum, analyses on *d’* were consistent with accuracy analyses and highlight the fact that content appears to bias a source decision.

Thus, the congruency effect for source decisions suggests such decisions were made using the content and not via an episodic retrieval process. For example, if an item was judged more likely to be a tweet because of content or stylistic elements, participants might have indicated it was from Twitter via a plausibility judgment or educated guess. To examine this, we examined source judgments for false alarms to foils (i.e., items incorrectly recognized as old), for which a source judgment would reflect a guessing strategy. We conducted 2 × 2 ANOVA with content (new headlines versus new tweets) and age as factors. Data from 27 younger and 23 older adults were included in the analyses, due to missing data from some participants. The source of non-studied tweets (*M* = 0.82, *SE* = 0.04, 95% CI [0.74, 0.90]) was identified more accurately than that of non-studied headlines (*M =* 0.67*, SE* = 0.04, 95% CI [0.59, 0.76], *F*(1, 48) = 5.58, *p* = .02, partial *η*^2^ = 0.10. This suggests that some linguistic structure or content makes tweets more easily identifiable as coming from social media, whereas the source of headlines might be less obvious. Interestingly, older adults were more likely to correctly identify the source of tweets and headlines (*M* = 0.85, *SE* = 0.04, 95% CI [0.77, 0.92]) than younger adults (*M* = 0.65, *SE =* 0.04, 95% CI [0.58, 0.72], *F*(1, 48) = 13.99, *p* < .001, partial *η*^2^ = 0.23. There was no interaction of content and age, *F* < 1.0.

To further determine whether the tweets and headlines differed in the extent to which source could be inferred solely from content, we examined whether naïve participants were successful at recognizing the source of the items when they were presented with no identifying (or contradictory) formatting information. Forty-one participants recruited on Amazon’s Mechanical Turk platform were presented the stimuli one at a time (content only, with no formatting) and asked to indicate whether the items were news headlines or social media posts (ratings are missing for two headlines; one was omitted due to programming error and one was included twice). Additional details on this study are reported in Additional file 1. Analyses by items revealed that the source of tweets (*M* = 0.89, *SEM* = 0.02) was identified more accurately than the source of headlines (*M* = 0.83, *SEM* = 0.02), *t*(156) = 2.04, *p* = .04, *d* = 0.35. Thus, both experimental and naïve participants were able to identify the source of tweets more accurately, likely due to content and linguistic cues present in the tweets.

Finally, to examine whether pre-experimental experience with social media or news sites and education affected how well participants recognized the items or their source, we tested correlation between measures of social media and news use and memory performance. Because age was significantly correlated with social media use, *r*(57) = −.62, *p* < .001, news reading, *r*(71) = .49, *p* < .001, and education, *r*(71) = .57, *p* < .001, we controlled for age in the tests of correlation. None of the correlation values were reliable, all *p* values > .07, suggesting that none of the measures predicted item or source memory performance.

## Discussion

To explore the factors that affect source and item memory for social media and news items, younger and older participants studied tweets and headlines that were formatted to look like items found on a Twitter feed or on CNN’s online platform. No age-related declines in item recognition performance were seen; however, consistent with Mickes et al. (2013), item memory was better for social media content than news content, regardless of the format. Thus, we replicated their observed advantage for social media content across age groups and using stimuli from a different social media platform. Overall performance was quite high and equivalent across age groups. Younger adults also showed an advantage for social media content on the source memory test. However, both age groups were negatively affected by a mismatch between content and format, suggesting that incongruency between these factors impairs source memory.

Regarding item memory, formatting information that indicated source of the material had no effect on performance. One hypothesis was that source information might have affected the encoding process, if social media posts were perceived as less relevant or important and afforded fewer attentional resources. User-generated sources are rated as less credible than other sources (Miller & Kurpius, 2010) and this may affect retention. Thus, tweets that were clearly identified as such might have been afforded less attention and remembered more poorly. However, this did not appear to be the case - social media content was remembered well, regardless of perceived source.

The fact that a similar memory enhancement effect was found for tweets as for Facebook posts and comments from news and entertainment sections (Mickes et al., 2013, experiments 1 and 3, respectively) extends the social media advantage to another form of social media. Several aspects of social media might affect memorability. Because of their nature, as noted by Mickes et al. (2013), these posts tend to be more intrinsically gossipy (i.e., they convey information about other members of a social group; e.g., Kurland & Pelled, 2000). Twitter posts might not have elicited such an effect, possibly because tweets have a less gossipy/social function than personal Facebook posts. To examine whether these factors might explain the mnemonic advantage of tweets over headlines, which are also outward-reaching but possibly less gossipy and more factual in nature, we collected additional data using an online platform to obtain estimates of the perceived levels of gossipy content in our headlines and tweets (additional information on the study is available in Additional file 1). Forty-two participants recruited via Amazon’s Mechanical Turk (none of the participants completed the source judgment task described above) rated each headline and tweet on how gossipy they perceived it on a scale from 1 (*not at all gossipy*) to 7 (*very gossipy*). Participants read the following definition of gossipy: “Gossip refers to the general sharing of details of other people’s lives, such as casual or unconstrained conversation or reports about other people, typically involving details which are not confirmed as true (according to the *Oxford Dictionaries*).” Stimuli were presented in random order and participants responded at their own pace. As noted above, ratings for two headlines were missing. On average, tweets were rated as more gossipy (*M* = 2.97, *SEM* = 0.08) than headlines (*M* = 2.74, *SEM* = 0.08), *t*(156) = 2.06, *p* = .04, *d* = 0.33. Overall, perceived level of gossipy content appeared to be low; however, the higher ratings for social media posts than news headlines does suggest that this specific aspect of the content might be influencing retention.

Mickes et al. (2013) suggested that another factor that made social media posts so memorable was the way they reflected natural language, in terms of both form and content. For example, a post on social media might be less edited and more spontaneous than other forms of writing, which might in turn make it more accessible to the reader, who will then remember it better. Tweets, because of the length restriction, might require more editing, becoming less spontaneous or less similar to natural language. Although the constraints of Twitter might involve more editing, this did not appear to eliminate the effect. Conversely, it is possible that even after the self-editing process, tweets still might have some natural language aspects that make them memorable.

The age-invariance of the item memory performance further extends Mickes et al.’s (2013) findings. Tweets might be less relevant to older adults, who use social media less than younger adults, and this demographic may view the information as less credible. Participants devote fewer attentional resources to less credible information and thus do not encode it as well. For example, the source of information affects false memory rates (Fenn, Griffin, Uitvlugt, & Ravizza, 2014): when participants encoded information on a Twitter platform or a non-social media platform, those exposed to the non-social media platform had greater confidence for recognizing false information. This suggests that knowing a source is less credible may cause individuals to engage in less effort to remember the content. This might be particularly pronounced in older adults because they are less likely to use Twitter and therefore may simply discredit the information or pay less attention to it. However, the robust memory performance for social media across ages suggests that the effect is not driven by levels of personal interest or relevance.

The most noteworthy contributions of the present work are the source memory findings. Older adults performed worse than younger adults did overall, consistent with prior reports (e.g., Balota et al., 2000; McIntyre & Craik, 1987; Spencer & Raz, 1995); however, their source memory deficit was largely due to very poor performance on incongruent items. Younger adults had an advantage in source memory for tweet content, possibly because it is more personally relevant and may activate more personal knowledge, thus resulting in a more elaborate memory trace. Thus, for this age group, the social media advantage reported by Mickes et al. (2013) also extended to source memory. For older adults, the lack of personal relevance and familiarity for tweets may have resulted in poorer memory for contextual details such as source when content and format mismatched. This suggests that a reader’s personal experience with the information might affect source memory. Our results did not fully support this hypothesis, however, because neither social media use nor news use predicted performance.

A key finding in the present study was the robust congruency effect in source memory. To remember an item (i.e., the content) and its origin (i.e., format), this information must be bound during the encoding phase (Raj & Bell, 2010). Thus, retrieval of specific details encountered during the study phase is necessary for correct source judgments. One explanation for the congruency effect may be the additive relationship between content and format. Craik and Tulving (1975) proposed that congruent items produce enhanced semantic elaboration, which in turn increases memory for that item during testing. Congruent events have a semantic relationship that prompts elaboration (integration into memory) during the encoding phase. For example, Staresina, Gray, and Davachi (2009) found that congruent items resulted in better memory for items and item-colors relative to incongruent items. However, if such congruency effects depend on effortful encoding strategies or attentional resources, older adults, who show deficits in self-initiated strategy use (Craik & Byrd, 1982; Skinner & Fernandes, 2009), might be less likely to use such factors. Indeed, older adults need additional environmental support to engage in more effortful strategies (Hay & Jacoby, 1999).

Older adults’ sensitivity to the effects of congruency suggests they might have relied on mechanisms such as plausibility judgments, including factors such as content or language, that might have served as cues for the source. Additional experience with language might support older adults in making such judgments or recognizing particular stylistic differences between social media and traditional news. Such a finding is consistent with evidence that older adults have richer semantic schemata that they can rely on (Umanath & Marsh, 2014). The high levels of source memory for congruent items does suggest that older adults can rely on this knowledge and do so accurately, as long as the content and source information (i.e., format) are congruent. The source judgments for foils further indicate that older adults are able to make educated guesses about where an item came from. Thus, the fact that older adults perform as well as younger adults for congruent items, which is highly atypical in the literature, reflects the underlying errors older adults make: They rely more heavily on content than formatting information that was available at encoding. Thus, they have higher rates of hits and lower rates of false alarms for congruent items (i.e., they rarely attribute a tweet presented as a Twitter post to CNN or a headline presented as a CNN post to Twitter) relative to younger adults.

However, this reliance on knowledge or sophisticated guessing of the source could have significant costs if individuals make determinations about the source of information based solely on content, because source is a key indicator of information validity or credibility. Although older adults appeared to have similar source memory to younger adults for congruent items and the age effect in the analyses on correct source identification was relatively small, when response bias was taken into account in the analyses on *d’*, their memory for source was significantly lower than younger adults. Older adults seem to be relying more on the content, rather than the format, as their incorrect attribution of source for incongruent items was much higher than in younger adults.

We also acknowledge that there may be differences in how one decides whether an item is “old” or “new,” which could subsequently affect the accuracy of source judgments for items declared to be old. Starns, Hicks, Brown, and Martin (2008) propose that source memory accuracy can vary with bias (liberal or conservative) to respond “old” in recognition decisions. In other words, bias can increase or decrease the proportion of items that are subjected to a subsequent source memory test. An individual with a liberal decision criterion would be more likely to declare items to be old even despite having weak memory for items. Because items that are identified as old must also undergo a source memory test, participants may show lower source accuracy simply due to the weaker memory signal. Conversely, participants with a conservative decision criterion would be less likely to identify items as old, due to selecting items only when there is a strong memory trace. Thus, source accuracy with a conservative decision criterion will be high. It is possible that older and younger adults varied in their decision criterion, meaning that the difference in source memory between the two groups could be strengthened or attenuated. In particular, older adults might have adopted a more liberal response criterion, as indicated by the slightly higher false alarm rates to non-studied foils. However, analyses on beta, a measure of criterion, did not indicate any age difference or effects of source or interaction. These analyses are included in Additional file 1.

### Limitations

We acknowledge three important limitations in the present study: the use of different presentation rates during encoding, the timing of the social media questionnaire, and the confound between stimulus type and font size. The different presentation rate was intended to equalize performance across ages due to generalized slowing in older adults (e.g., Salthouse, 2004). Furthermore, Frieske and Park (1999) found that a significant portion of variance in recall and source recognition was accounted for by processing speed. In addition, in naturalistic settings, individuals might self-regulate how long they spend examining an item on social media or the news; thus, we assumed that older adults might spend more time than younger adults reading the material. Although the different presentation rates were motivated by both theory and prior empirical work, this choice did generate potential costs. First of all, the results obtained might be less generalizable to other studies or situations where presentation rate is held constant or individuals have no control over study time allocations. Second, the absence of item memory deficits might be an artifact of this manipulation. Future studies should examine whether the equivalent performance on item memory persists when study time is not confounded with age or allow a self-paced presentation rate to more closely mimic a truly naturalistic experience. However, given the poor performance of older adults in the source task, it is possible that reduced time would result in even poorer performance.

A second limitation concerns the administration of the social media and news-use questionnaire. Participants completed this measure as a filler task before the recognition test. The items on this questionnaire might have biased participants to attend differentially to the different items on the test. Although this is a valid concern, it is worth noting that the formatting of the items during the encoding phase made the manipulation of item type quite explicit. Because of pre-existing associations and differential experience with the types of content and the two different sources, re-directing participants’ attention to the fact that the stimuli came from social media or news sources might have generated some bias in responses. Although we cannot rule this out, there are two lines of evidence that make such a concern less likely. First, if the questionnaire in some way biased participants to differentially attend to information of one type or another, such effects might have emerged in item memory, such that older adults, who preferentially attend to news over social media, would show a reversal of the mnemonic advantage of social media content. As noted above, this did not occur. Such a bias might manifest on the source memory test, such that older adults would preferentially attribute content to the news source and younger adults to the social media source. However, this did not seem to be the case. A second line of evidence suggesting that the timing of the questionnaire had a limited impact comes from research on false memories. The popular Deese-Roediger-McDermott paradigm for eliciting false memories involves presenting lists of words that all converge on one, non-studied critical lure (Roediger & McDermott, 1995). This lure is often recalled or recognized at high rates. Warnings about the nature of the lists can reduce the illusion but not eliminate it. Importantly, warnings are most effective when administered prior to the encoding phase, with little to no effect of warnings when they are administered between encoding and retrieval, especially in older adults (McCabe & Smith, 2002). This suggests that once information is encoded, it is difficult for decision strategies to influence memory performance.

Confounding by font size, as noted in “Materials”, was intended to maintain a more naturalistic presentation. It is possible that different stimulus sizes might have affected performance; specifically, older adults might have had more difficulty with the smaller font size of the tweets due to deficits in perceptual processes (Baltes & Lindenberger, 1997). Given the overall lack of age effects in item memory, this seems not to have occurred. Furthermore, because stimuli at test were presented in a novel font that was constant across items, it seems unlikely that it would affect the congruency effect. Although there is some evidence for a font-size effect (e.g., Mueller, Dunlosky, Tauber, & Rhodes, 2014; Rhodes & Castel, 2008), it primarily affects judgments of learning and has no effect on actual performance.

## Conclusion

Remembering where a piece of information was seen can be difficult. The negative effects of a mismatch between content and format might be particularly relevant as traditional and social media sources become more integrated. Because up to 67% of individuals report obtaining news from social media sources (Shearer & Gottfried, 2017), the “blending” of news sources might make it harder to distinguish between news and social media. If the source of news items encountered in a social media platform is poorly remembered, this might result in an inability to locate the item at a later date. To the extent that the content was perceived as more “news-like” or “social media-like” - regardless of actual source - a subsequent search for that item might fail, thus affecting one’s ability to retrieve that item. Incorrectly attributing information to a credible or non-credible source could have potential consequences for how that information is evaluated and used in other contexts. Our results suggest that older adults in particular might be susceptible to source errors because of their reliance on content for making source decisions.

## Supplementary information

**Additional file 1.** Supplemental Materials.

## Data Availability

Data will be available upon request from the corresponding author (JHC).

## Footnotes

1 The number of older adults recruited was determined based on the number of participants able to participate during the summer session (younger adults were tested during the spring semester). Data collection was suspended after the summer to minimize differences in familiarity to the news items or tweets. As indicated by the power analysis, 32 participants exceeded the minimum number of participants necessary to detect an effect.

2 A separate power analysis using a moderate effect size of 0.50 (under the assumption that the difference between tweets and headlines might not have been of the same magnitude) indicated a total sample of 54 was necessary to obtain 0.95 power.

3 We acknowledge that the different time scales on the measure were not ideal and reflect an error on the part of the experimenters.

4 We thank an anonymous reviewer for suggesting this analysis.

5 If participants disregarded or failed to encode the formatting information, they could still correctly recognize Tweets and Headlines being studied in a Twitter format or a CNN format via a response strategy (bias) in which they declare that they recognized Tweets as presented in Twitter format and Headlines as presented in CNN format. This would not reflect source memory, but a bias to select the format that corresponded to the item’s content. For congruent items, source memory accuracy would be very high, as Tweets, because of their content, would be attributed to a Twitter format (and vice versa for headlines). Conversely, errors (i.e., false alarms) in which Tweets were incorrectly attributed as having been in a CNN format would be low, due to the content/format mismatch. Therefore source memory would appear to be good as d’ would be high. However, in such an analysis, there is no source memory, just a bias (i.e., successful matching strategy) to pick the source that matches the formatting characteristics of the item’s content. For incongruent items the hit rate would be low as the Tweets would be logically, but incorrectly, attributed as having been presented on Twitter backgrounds. In this condition, the false alarm rate would be very high because tweets would be incorrectly attributed to have been presented on a Twitter background. Therefore, we calculated d’ as described above in order to take into account for this response bias strategy.
