# Supplementary material for: Reading the news on Twitter: Source and item memory for social media in younger and older adults
Source: Cogn Res Princ Implic. 2020 Mar 14;5:11. doi: 10.1186/s41235-020-0209-9 (PMC7072077; doi:10.1186/s41235-020-0209-9)
Supplement: Supplementary file 1 — Additional file 1. Supplemental Materials. [file 41235_2020_209_MOESM1_ESM.docx]

**Supplemental Materials**

Reading the News on Twitter:

Source and Item Memory for Social Media in Younger and Older Adults

Kimberly A. Bourne, Sarah C. Boland, Grace C. Arnold, and Jennifer H. Coane

Colby College

Address correspondence to:

Jennifer H. Coane, PhD

Department of Psychology

Colby College

4000 Mayflower Hill Drive

Waterville, Maine, 04901

United States of America

[jhcoane@colby.edu](mailto:jhcoane@colby.edu)

*Author note*: Portions of these data were presented at the 2015 meeting of the Eastern Psychological Association in New York, NY. This research was supported in part by Understanding Human Cognition grant #220020426 from the James S. McDonnell Foundation awarded to JHC.

Kimberly Bourne, Department of Psychology, Colby College; Sarah Boland, Department of Psychology, Colby College; Grace Arnold, Department of Psychology, Colby College; Jennifer Coane, Department of Psychology, Colby College.

**Table of Contents**

**Stimuli 3**

**Norming Studies 7**

**Accuracy Analyses of Item Memory 9**

**Source Identification 2-way Interactions 9**

**Source Identification Analyses on Beta 10**

**Study Materials**

| **Item Type** | **Item** |
| --- | --- |
| Headline | Decline in smoking rates could increase deaths in lung cancer |
| Headline | Stan Lee draws Spider-Man for a child with autism |
| Headline | Alabama governor sorry for police incident, officer pleads not guilty |
| Headline | How a profoundly disabled boy became a running champ |
| Headline | Unreasonable mother' shapes a world that embraces disabled children |
| Headline | Scope superbug: How long did the FDA know about the problem? |
| Headline | Early consumption may prevent peanut allergy, new study suggests |
| Headline | Amanda Knox case: Court to rule on her conviction for roommate's murder |
| Headline | How right was Romney? 2016 campaign would hinge on American buyer's remorse |
| Headline | Obamacare at the Supreme Court: Justices again divided over landmark health law |
| Headline | The island where people forget to die |
| Headline | Why would you want to be a cop? |
| Headline | Is U.S. Air Force superiority under threat? |
| Headline | Four simple steps to beating the holiday blues |
| Headline | Saudi Ambassador to U.S. won't rule out building nukes |
| Headline | Somalia hotel attack: Diplomat, others killed |
| Headline | Germanwings co-pilot Andreas Lubitz declared 'unfit to work,' officials say |
| Headline | Happy eating: Ingredient to a long life, in your cereal bowl |
| Headline | Early consumption may prevent peanut allergy, new study suggests |
| Headline | Teen goes to Target looking for a tie, gets that and more |
| Headline | Fit Nation: Down, but not out, in 20 seconds |
| Headline | Sean Penn and others rile up social media at the Oscars |
| Headline | St. Louis officer under fire for turning off dashcam video during arrest |
| Headline | Plague blame game: Gerbils replace rats as prime suspects |
| Headline | Shatner defends absence from Nimoy's funeral |
| Headline | Chimps still stuck in research labs despite promise of retirement |
| Headline | U.S. tourists arrested for carving initials into wall at Rome's Collosseum |
| Headline | 28 Internet acronyms every parent should know |
| Headline | Activist: ISIS holds 150 Christian hostages, will threaten to kill them |
| Headline | Kanye West apologizes to Beck, Bruno Mars |
| Headline | The latest Obamacare challenge: What you need to know |
| Headline | Civil Rights groups nervous about Supreme Court case on housing discrimination |
| Headline | Egypt unveils plan to build glitzy new capital |
| Headline | Invisible threat to maternal and child health |
| Headline | Minister Creflo Dollar asks for $60 million in donations for a new jet |
| Headline | Why liberals should get behind marriage |
| Headline | Former 'America's Next Top Model' contestant slain |
| Headline | Army report: Bergdahl intended to walk to nearest base |
| Headline | Hubble captures triple solar eclipse on Jupiter |
| Headline | Hush! There's a secret bar inside this bar |
| Headline | Lupita Nyong'o's $150,000 pearl Oscars dress stolen |
| Headline | Alaska flights canceled over ash from Russian volcano |
| Headline | Jury finds Eddie Ray Routh guilty in 'American Sniper' case |
| Headline | Rare birth: Baby born completely encased in amniotic sac |
| Headline | Nigeria: Boko Haram bomb factory uncovered in troubled northeast |
| Headline | The health risks of cyberbullying in College |
| Headline | Mad about free speech and North Korea? Hack back, says group |
| Headline | 6 ways to improve odds and beat heart disease |
| Headline | Authorities: three men attempted to join ISIS, had ambitious plans |
| Headline | Kofi Annan: Leaders in Davos must act now to confront global issues |
| Headline | Meet the 10-year old maths genius who's just enrolled in college |
| Headline | Mexican tour boat hits whale, leaving Canadian woman dead |
| Headline | MH370: 21st century technology might have solved the mystery |
| Headline | Southwest planes, grounded over missed inspections, allowed to fly |
| Headline | Former Mets VP: Why sports needs women in management |
| Headline | Obamacare aide Marlon Marshall departs White House ahead of 2016 |
| Headline | Photo of kids climbing on a Vietnam memorial draws anger |
| Headline | What will happen if the bees disappear? |
| Headline | Woman suing California for her right to die at home |
| Headline | World's most dangerous footpath set to reopen in Spain |
| Headline | Delta flight skids off LaGuardia runway, stops feet from frigid waters |
| Headline | Stunning geometric shapes appear in snow on frozen, isolated lakes |
| Headline | Harrison Ford 'battered, but ok' after small-plane crash, son says |
| Headline | Lake Nicaragua: See this massive lake now before it's changed forever |
| Headline | Hundreds of skeletons found under Paris supermarket |
| Headline | From dashing spy to Vogue illustrator: The double life of Mr. Stonehouse |
| Headline | CPAC 2015: GOP White House contenders unite against Hillary Clinton |
| Headline | Divided House GOP turns to special rule to pass budget |
| Headline | Family Matters' Darius McCrary busted for failure to pay child support |
| Headline | The fastest plane in the world |
| Headline | Kerry Washington brings cheering crowd to its feet in GLAAD speech |
| Headline | LGBT people in rural areas struggle to find good medical care |
| Headline | Why are these NFLers retiring early? |
| Headline | Powerful solar storm sparks stunning aurora around the world |
| Headline | I am the proud son of an immigrant' |
| Headline | Woman charged with attempted murder in Colorado fetal abduction case |
| Headline | Watch March Madness at work? |
| Headline | Learn to live with it: Becoming stress-free |
| Headline | Ancient Egyptian tomb uncovered |
| Headline | Work/life balance an impossible dream? |
| Tweet | I so wish "The Theory of Everything" was called "Look Who's Hawking" |
| Tweet | The dress conversation is seriously going to make me take a Xanax. |
| Tweet | I accidently recorded the Gmod loading screen for 26 hours. |
| Tweet | I like my men like I like my coffee. Silent. |
| Tweet | Just found out that I have lived exactly 10,000 days today. Wow |
| Tweet | Enrollment in synchronized swimming classes drastically increases post new Avengers Age of Ultron trailer. |
| Tweet | be the person your dog thinks you are |
| Tweet | I don't know what to say on Twitter no mo |
| Tweet | Love a good cable knit blanket |
| Tweet | Just lost giant light wall privileges for giving up the jig. Idiot. |
| Tweet | Watching Real Steel. the definition of a #GuiltyPleasure |
| Tweet | Sometimes you have to make hard choices in order to find your true happiness |
| Tweet | Daddy… will the icloud eventually block out the sun? |
| Tweet | Just watched The Jinx finale and cannot process thought or emotion. |
| Tweet | My bedroom clock is right again |
| Tweet | I just ate two chipotle burritos and immediately fell asleep |
| Tweet | No campaign has ever been less pitched at me than #noceilings |
| Tweet | You know you're in my car when my iPod switches from Katy Perry to Marilyn Manson |
| Tweet | I love Mexico! How come I have only travelled here now! |
| Tweet | True Life: My Neighbor Is An Aspiring Opera Singer |
| Tweet | Bleach ya brows and call it FASHUN. |
| Tweet | Me and my wife met with President Obama two weeks ago |
| Tweet | Amazing. Beck and Chris Martin sure did… stand there super well. So much Energy! |
| Tweet | "Cool" is ever evolving. Don't try to keep up, just relax. |
| Tweet | Hmmm. Jamie Foxx reading braille next to Stevie Wonder. Not sure how I feel about that. |
| Tweet | I live at the end of a 5 and a 1/2 minute hallway |
| Tweet | Los Angeles WAKE UP! Where you at!!! I'm coming to EL RAY THEATRE |
| Tweet | Miami, Get to wynwood walls now. |
| Tweet | Shia Lebouf sure knows how to read a teleprompter. |
| Tweet | Someone fill me in on this dress thing. I'm so confused |
| Tweet | Usher can sing. But did that harpist come from the Ariana Grande school of hair design? |
| Tweet | Getting new tools to the people fighting Ebola is harder than it needs to be |
| Tweet | Happy Birthday for the 113 years of success |
| Tweet | How was there not a "who wore it better" posted yet from last night's show? |
| Tweet | Microbes-not missiles-are what could kill 10M people |
| Tweet | North Carolina still has my heart after all these years |
| Tweet | (Whatever, I looked good with henna tattoos and crimped hair) |
| Tweet | Some things of this world are indigestible |
| Tweet | My birthday is tomorrow and I've never been so excited for a new year of LIFE |
| Tweet | It's official. Katy Perry is magic. |
| Tweet | I kinda like that this winter storm is call Thor. |
| Tweet | Everyone tweet a picture of yourself with water! Drinking, showering, cooking, cleaning… |
| Tweet | Happy Mothers Day Mamas x |
| Tweet | Just watched Nightcrawler after months of being excited and it WAS AMAZZING! Go and see it! |
| Tweet | The American Candy tour kicks off in one month from today! |
| Tweet | how many times a day do you have to charge an Apple Watch? |
| Tweet | I must say: Austin, Texas is a very cool town. |
| Tweet | I can't tell you often that chef that look likes Phyllis Diller will just pop into my head |
| Tweet | Curled up on the couch watching my love on #ChicagoFire I love this. |
| Tweet | Oh, my, I'm enjoying this Cavs-Spurs game. |
| Tweet | You might as well get it while you can, babe. cause you know you ain't getting any younger |
| Tweet | I can't stop thinking about Jade and Jade's dad. |
| Tweet | Just unfollowed Cheesecake Factory |
| Tweet | If you ever want to feel like chic-free troll, visit Intelligentsia in Silverlake |
| Tweet | India carney is dope |
| Tweet | I just saw @playgoeswrong It is incredibly funny. And it was sold out. |
| Tweet | That peanut butter + low carb tortilla life |
| Tweet | Up too late talking about Robert Durst and you should be too tbh |
| Tweet | And here I thought algebra served no purpose |
| Tweet | Is it Ben or is it Fleur? Tonight we are going to find out. |
| Tweet | Georgia and her sisters celebrated their bday last night |
| Tweet | How many times is an alarming number of times to have Googled "eye contact"? |
| Tweet | It's good to be the king. It sucks to be the czar. Unfair, but so it goes. |
| Tweet | Really proud of my brother and his new album. |
| Tweet | At least Russia's never boring. *nervous laugh* |
| Tweet | The silence depressed me. It wasn't the silence of silence. It was my own silence |
| Tweet | Drove around bev hills for an hour looking for a good sunset spot, missed the sunset… |
| Tweet | About to call some people who preordered #AmericanCandy to say thx I luv u |
| Tweet | Well that's all the time we have…Until next time yall be safe! |
| Tweet | How can so many young attractive people be murderers? #the following |
| Tweet | It just occurred to me that Food Pornographer is a legit job title now. |
| Tweet | Rainy day in the desert |
| Tweet | Thank you to everyone who voted. We're thrilled! |
| Tweet | And I wanted to say hello to everyone who follows me. |
| Tweet | Finally getting to spend a little time with my daughter today.. |
| Tweet | And now it's time to say goodnight to everyone. Or good morning. |
| Tweet | And Rebel Wilson is a total star |
| Tweet | So sad to hear the news from Pakistan. Rest in peace. |
| Tweet | But really sad to see Robin Williams. Realise how much he is missed. |
| Tweet | You know winning isn't important to me but if I do win I will rescue some kittens |

**Norming Study 1 – Source Judgment Task**

The goal of this study, conducted after the original experiment, was to determine whether there were differences in the accuracy with which participants could determine the actual source of the stimuli used. Because stimuli were naturalistically derived (i.e., they were real tweets or headlines, collected online in Spring of 2015), there are likely systematic differences in linguistic style, content, and punctuation, for example, as well as content differences. Participants were recruited via Amazon Mechanical Turk (M-Turk) Participation was restricted to M-Turk users with a minimum of a high school education, a United States IP address, and a prior approval rating of 95%.

*Participants*

Forty-one participants completed the task. The mean age was 33.61 (*SEM* = 1.40, range = 21-55). Eighteen participants identified as female and 23 as male. The majority of participants had some college education (n = 11) or had completed college (n = 23). Three had some graduate education or had completed graduate school and four reported a high school degree as the highest level of education attained. Participants were compensated $1.50.

*Materials and Procedure*

The task was administered using the software platform Gorilla (gorilla.sc). The 160 stimuli used in the main experiment were used (however, due to experimenter error, one stimulus was omitted and one was presented twice; analyses exclude the latter). Participants read the following instructions:

*You will be shown a number of statements. Some of them are headlines and some are social media posts. To the best of your ability, indicate whether you think each individual statement comes from social media or from a reputable news source. We are interested in subtle linguistic differences between social and formal media. Please use your best judgment and do not consult external sources.*

The stimuli appeared one at a time, in random order, in black font on a white background. Responses were made by clicking on one of two buttons presented on the screen that appeared below each statement. The two buttons were labeled as “News Headline” and “Social Media Post.” Once participants clicked on one of the buttons, the program automatically advanced to the next trial. The task took on average 15 minutes.

*Results*

Because we are interested in how individual items were identified as coming from a news source or a social media source, we report item analyses on the proportion of correct judgments. The analyses included 80 tweets and 78 headlines, due to the error noted above. Tweets (*M* = .89, *SEM* = .02) were identified as social media posts more accurately than headlines (*M* = .83, *SEM* = .02) were identified as news items, *t*(156) = 2.04, *p* = .04, *d* = .35. There was no difference in the speed to make correct decisions, t < 1.0 (*M_tweet_* = 3184.32, *SEM* = 249.12; *M_headline_* = 3601.02, *SEM* = 346.78).

**Norming Study 2 – Ratings of “Gossipy” Nature**

In a second norming study, also conducted after the original experiment, we examined whether tweets and headlines differed in how “outward-reaching” or gossipy they were. Participants were recruited from M-Turk using the same restrictions as in Norming Study 1. Participants who had completed one task were not eligible for completing the other.

*Participants*

Forty-two participants completed the task. The mean age was 34.95 (*SEM* = 1.51, range = 19-58). Twenty-two participants identified as female and 20 as male. Twenty-five reported having completed college, five had completed or attended graduate school, eight reported some college, and four reported high school as the highest level of education. Compensation was set at $1.50.

*Materials and Procedure*

This task was also administered using Gorilla. Participants rated the experimental stimuli in terms of how gossipy they perceived them to be. The following instructions were presented:

*Gossip refers to the general sharing of details of other people’s lives, such as casual or unconstrained conversation or reports about other people, typically involving details which are not confirmed as true (according to the Oxford Dictionaries). Please rate the extent to which each of the following statements is gossipy according to this definition. A rating of 7 indicates the statement or comment is very gossipy – it shares many details about the author’s or someone else’s life; a rating of 1 indicates the statement or comment is not at all gossipy. There are no right or wrong answers; we are interested in how different individuals perceive the content of various statements from different sources.*

The stimuli were presented one at a time in random order. Below the stimulus, a Likert scale from 1-7 was presented. The anchors were *not at all gossipy* and *very gossipy.* After participants clicked on their selected response, the program automatically advanced to the next stimulus. The task took approximately 15 minutes to complete.

*Results*

As in Norming Study 1, we report item analyses. For each item, we calculated the mean gossipy rating across participants; higher means reflect a more gossipy nature than lower means. Although overall the stimuli tended to be perceived as low in gossipy qualities, tweets (*M* = 2.97, *SEM* = .08) were rated as more gossipy than headlines (*M* = 2.74, *SEM* = .08), *t*(156) = 2.06, *p* = .04, *d* = .33. There was no difference in response latencies, *t* < 1.0, (*M_tweet_* = 3341.47, *SEM* = 198.49; *M_headline_* = 3245.41, *SEM* = 173.97).

**Accuracy Analyses of Item Memory**

To analyze item memory, a 2 (age) x 2 (content) x 2 (format) mixed ANOVA was conducted on the proportion of correct responses (e.g., correctly saying a studied tweet or headline was old; see Supplemental Table 1 for means). We found a main effect of content, *F*(1, 72) = 13.64, *p* < .001, partial η^2^ = .16, such that tweets (*M* = .81, *SE* = .02, 95% CI [.72, .79]) were recognized better than headlines (*M* = .76, *SE* = .02, 95% CI [.78, .85]), regardless of format. No other effects were reliable, all *Fs* < 1.02, all *ps* > .36.

False alarm rates were analyzed in a 2 (age) x 2 (content) mixed ANOVA. No effects were significant, all *F*s < 2.4, *p*s > .12.

Supplemental Table 1

*Average Proportion of “Old” Responses as a Function of Age, Item Content, and Item Format (Standard Errors in Parentheses)*

|  | Older Adults | | | Younger Adults | | |
| --- | --- | --- | --- | --- | --- | --- |
|  | CNN format | Twitter format | New | CNN format | Twitter format | New |
| Headline | .76 (.03) | .75 (.03) | .13 (.02) | .76 (.03) | .76 (.03) | .08 (.02) |
| Tweet | .83 (.03) | .79 (.03) | .13 (.02) | .82 (.02) | .82 (.02) | .08 (.02) |

**Source Identification 2-way Interactions**

In the analyses on source identification, two 2-way interactions were reliable: The content by age interaction and the content by format interaction. These analyses are reported below.

When examining the content by age interaction, *F*(1, 72) = 10.09, *p* = .002, partial η^2^ = .12, follow-up analyses revealed that, overall, younger adults (*M* = .72, *SEM* = .02) outperformed older adults (*M* = .57, *SEM* = .02), *F*(1, 72) = 32.02, *p* < .001, partial η^2^ = .31. The source of Tweets (*M* = .67, *SEM* = .01) was identified correctly more often than the source of headlines (*M* = .32, *SEM* = .02), *F*(1, 72) = 12.43, *p* = .001, partial η^2^ = .15. However, a significant interaction, *F*(1, 72) = 10.09, *p* = .002, partial η^2^ = .12, revealed that the source identification advantage for tweets was only present in younger adults, who were more accurate at identifying the source of tweets (*M* = .76, *SD* = .12) than that of headlines (*M* = .67, *SD* = .13), *t*(41) = -4.58, *p* < .001. There were no differences in identification for older adults (*M_headline_* = .57, *M_tweet_* = .58), *t*(31) = -.28, *p* = .78.

The content by format interaction once again indicated that the source of tweets (*M* = .68, *SEM* = .02) was correctly identified more often that the source of headlines (*M* = .63, *SEM* = .01), *F*(1, 73) = 1.17, *p* < .001, partial η^2^ = .16. There was no effect of format, *F* < 1.0. However, as indicated by the interaction, *F*(1, 73) = 71.22, *p* < .001, partial η^2^ = .49, there was a large effect of format and content congruency on correct source identification. When items (i.e., tweet or headline) had been studied in a format congruent with their origin, source identification was significantly higher than when the format was incongruent. Congruent tweets (*M* = .83, *SEM* = .02) and congruent headlines (*M* = .78, *SEM* = .02) were correctly identified as coming from the studied source more than incongruent tweets (*M* = .53, *SEM* = .03) and incongruent headlines (*M* = .49, *SEM* = .03), *t*(73) = 6.67, *p* < .001 and *t*(73) = 6.96, *p* < .001, respectively. Thus, source identification appeared to be heavily influenced by the content, such that participants tended to attribute an item to the original source, not to the studied source.

**Source Identification Analyses on Beta**

To examine whether underlying differences in criterion (i.e., liberal vs. conservative responses) might have affected source memory accuracy, we conducted analyses on beta. These estimates were obtained using the same parameters as were used for calculating *d'.* An age by content ANOVA revealed no main effect of age, *F*(1, 72) = 1.38, *p* = .25, no main effect of content, *F* < 1.0, *p* = .43, and no interaction, *F* < 1.0, *p* = .92. Thus, it does not appear as if underlying differences in response bias were significantly contributing to the observed effects on accuracy in source identification.

Supplemental Table 2

*Source Memory Estimates of Bias (Beta) as a Function of Age and Item Content (Standard Error in Parentheses)*

|  | Younger Adults | Older Adults |
| --- | --- | --- |
| Headline | .89 (.09) | .76 (.10) |
| Tweet | .97 (.10) | .86 (.12) |
